# Pilot-scale microalgae cultivation and wastewater treatment using high-rate ponds: a meta-analysis

**DOI:** 10.1007/s11356-024-34000-7

**Published:** 2024-07-10

**Authors:** Sharon B. Velásquez-Orta, Isaura Yáñez-Noguez, Ignacio Monje Ramírez, María Teresa Orta Ledesma

**Affiliations:** 1 School of Engineering, Merz Court, Newcastle University, Newcastle Upon Tyne, NE1 7RU UK; 2 Instituto de Ingeniería, Universidad Nacional Autónoma de México, Av. Universidad 3000, C.P. 04510 Ciudad de México, Alcaldía Coyoacán México

**Keywords:** High-rate algal pond, Pilot scale, Meta-analysis, Microalgae, Outdoor cultivation, Wastewater

## Abstract

Microalgae cultivation in wastewater has been widely researched under laboratory conditions as per its potential to couple treatment with biomass production. Currently, only a limited number of published articles consider outdoor and long-term microalgae-bacteria cultivations in real wastewater environmental systems. The scope of this work is to describe microalgal cultivation steps towards high-rate algal pond (HRAP) scalability and identify key parameters that play a major role for biomass productivity under outdoor conditions and long-term cultivations. Reviewed pilot-scale HRAP literature is analysed using multivariate analysis to highlight key productivity parameters within environmental and operational factors. Wastewater treatment analysis indicated that HRAP can effectively remove 90% of NH_4_^+^, 70% of COD, and 50% of PO_4_^3−^. Mean reference values of 210 W m^−2^ for irradiation, 18 °C for temperature, pH of 8.2, and HRT of 7.7 are derived from pilot-scale cultivations. Microalgae biomass productivity at a large scale is governed by solar radiation and NH_4_^+^ concentration, which are more important than retention time variations within investigated studies. Hence, selecting the correct type of location and a minimum of 70 mg L^−1^ of NH_4_^+^ in wastewater will have the greatest effect in microalgae productivity. A high nutrient wastewater content increases final biomass concentrations but not necessarily biomass productivity. Pilot-scale growth rates (~ 0.54 day^−1^) are half those observed in lab experiments, indicating a scaling-up bottleneck. Microalgae cultivation in wastewater enables a circular bioeconomy framework by unlocking microalgal biomass for the delivery of an array of products.

**Graphical Abstract:**

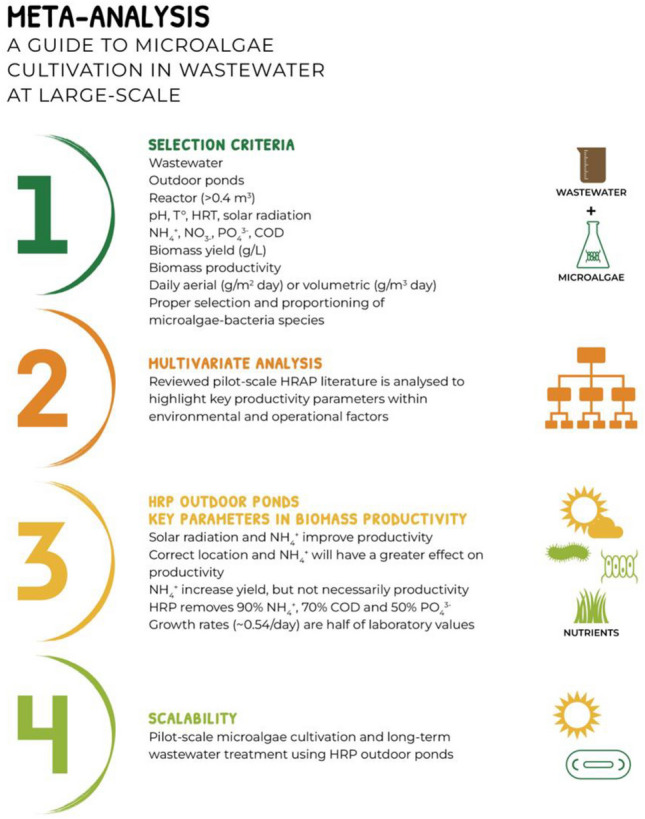

**Supplementary Information:**

The online version contains supplementary material available at 10.1007/s11356-024-34000-7.

## Introduction

During the last 10 years, the use of photosynthetic microalgae has retaken vigour in wastewater treatment research. This is shown by the exponential increase in the number of publications showcased in the literature (Li and Zhu 2021). This reached, in 2023, > 3000 in ScienceDirect and > 17,000 in Google Scholar. Elsevier Scopus database indicates that the main global interest falls in the depletion of carbon dioxide (CO_2_) and the production of biomass, lipids, and biofuels. Microalgal biomass, grown in wastewater, can have commercial uses, to generate products for the chemical, agricultural, or energy industry (Gong and Bassi 2016; Vassilev and Vassileva 2016; Sudhakar et al. 2019; Bhattacharya and Goswami 2020; Abdelfattah et al. 2023, Walls et al. 2019; González-Balderas et al. 2020a; González-Gálvez et al. 2021; González-Balderas et al. 2020b, c; Romero-Frasca et al. 2021; Montaño San Agustin et al. 2022; Vielma Hernández et al. 2022; Nava-Bravo et al. 2021). Within the chemical sector, recent applications include the production of bioplastics or biocomposites (González-Balderas et al. 2020b; López-Rocha et al. 2020; Cheah et al. 2023).

The potential of microalgae growth is well known, either under phototrophic, mixotrophic, or heterotrophic conditions. Most microalgae naturally grow under photoautotrophic conditions, as they require less energy. Using wastewater instead of the nutrient medium can significantly reduce microalgae cultivation costs, from 2.71 to 0.73 $/kg biomass (Kang et al. 2015), as it can completely replace the use of fresh water and fertilisers (Newby et al. 2016). Different types of wastewaters derived from the domestic, chemical, agricultural, and petrochemical industries have been reported to produce microalgal biomass (Gonçalves et al. 2017; Hernández-García et al. 2019; González-Gálvez et al. 2021; González-Balderas et al. 2020b; Valeriano González et al. 2020; González-Balderas et al. 2021; Romero-Frasca et al. 2021; Montaño San Agustin et al. 2022; Vielma Hernández et al. 2022; Nava-Bravo et al. 2021). Nutrient-rich effluents have also been used as a culture medium for microalgae, such as leachate, centrate, palm oil mill effluent, swine, and poultry wastewater, because of their high nitrogen and phosphorus content (Udaiyappan et al. 2017; Romero et al. 2018; Cheng et al. 2019; Hernández-García et al. 2019; Montaño San Agustin et al. 2022).

Photoautotrophic growth, in wastewater, at the laboratory scale has produced satisfactory results; however, the same microbial strains may not have a successful outcome at large-scale due to complex biological interactions between organisms and the environment. Recently, research has been steered towards understanding the limitations of pilot scale systems (here referred to as capacities > 0.4 m^3^). Scaling-up under outdoor conditions has so far highlighted the importance of microalgae polycultures, growing medium, and environmental conditions (Novoveská et al. 2016; Chew et al. 2018; 62. Schade and Meier 2019; González-Camejo et al. 2021).

Regarding microalgae polycultures, native species from wastewater are preferred as they are already acclimatised to local environmental conditions. The composition of microalgae-bacteria consortia constantly changes due to fluctuations in temperature, solar irradiation, nutrients, organic load, and pH, which are not homogeneous. Such conditions should be considered in studies using synthetic wastewater (Komolafe et al. 2014; Arango et al. 2016; Chist 2016; Zhang et al. 2016; Salama et al. 2017; Chew et al. 2018; Perera et al. 2019; SundarRajan et al. 2019); otherwise, results would not be comparable to real wastewater conditions.

**Fig. 1:**
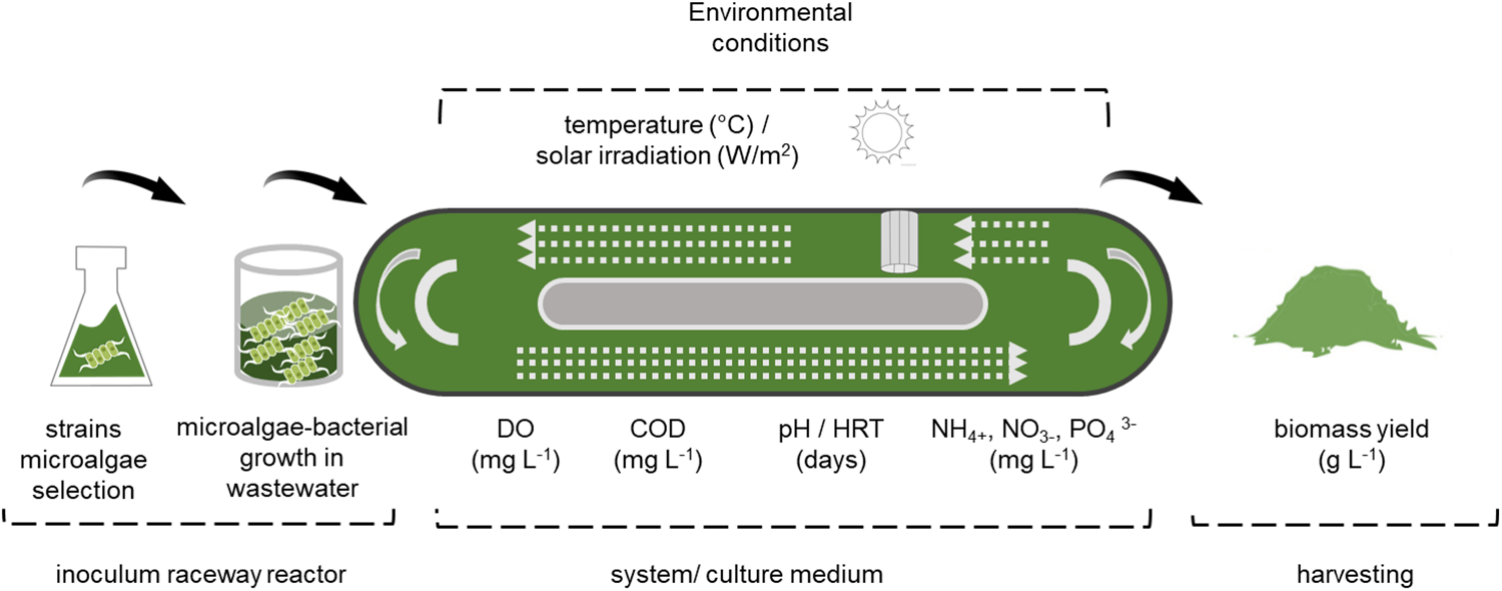
Scaling-up of microalgae cultivation and design/operation parameters affecting microalgae growth in HRAP. DO, dissolved oxygen; COD, chemical oxygen demand; pH, potential hydrogen; HRT, hydraulic retention time; NH_4_^+^, ammonia; NO_3_^−^, nitrogen nitrates; PO_4_^3−^, orthophosphates; T (°C), temperature (degrees Celsius); solar irradiation is Watts per square metre W m^−2^

Figure 1 shows the characteristics of HRAP and a pathway for scaling up. Production starts with cultivations lower than a litre; upscaling is then performed to have enough inoculum to initiate an open raceway system. A harvesting system is finally employed to separate water from the obtained biomass.

**Fig. 2:**
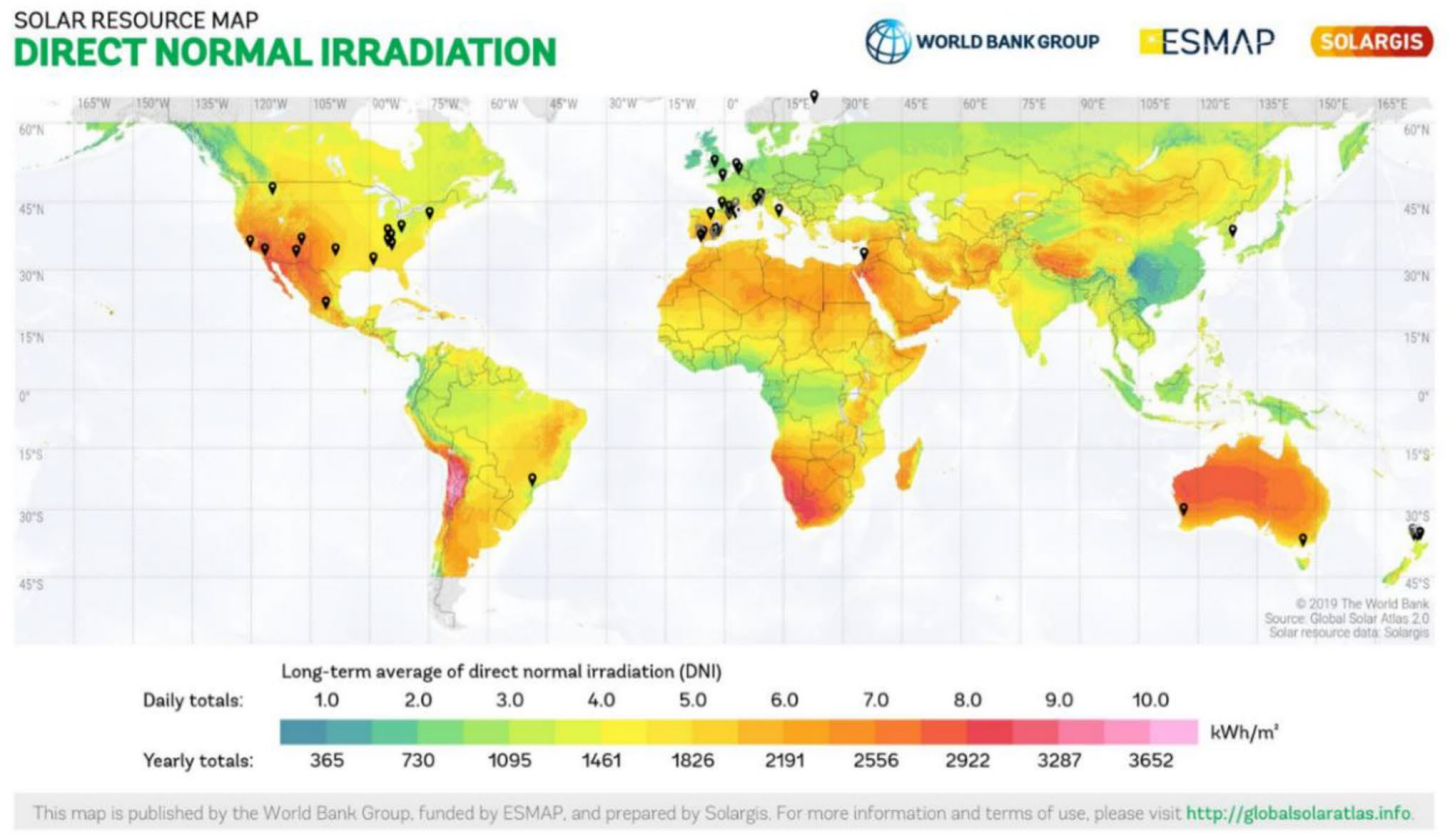
Location of different reported large-scale outdoor reactors for wastewater treatment using microalgae-bacteria consortium. Modified irradiation map in kWh m^−2^ taken from the worldwide electronic publication Solargis.info 2020—solar resource maps of the world in https://solargis.com/maps-and-gis-data/download/world. The raceway located in Mexico belongs to our research group

According to the Elsevier Scopus database (2022), around 200 studies related to microalgae cultivation in ponds using wastewater have been published in scientific journals. Most of them were performed in Spain followed by New Zealand and the U.S.A. It should be noted that if search parameters are further specified using words such as “photobioreactor”, “outdoor”, or “large-scale”, the documents obtained are less than 82. Thus, only a few studies have been documented regarding outdoor, long-term, microalgae-focused, wastewater cultivation. Figure 2 shows the location of documented outdoor reactors for wastewater treatment by microalgae-bacteria systems related to solar irradiation. Also, companies that apply this technology for commercial applications are represented in Table 1.

**Table 1:** Pilot-scale (up to 0.4 m^3^) microalgal biomass production using different types of wastewaters

Microalgae	System	Location	Link
Consortia	Algal biological reactor (ABR)	UK	https://i-phyc.com/the-iphyc-solution/
Consortia	Photobioreactors	USA	https://www.clearassolutions.com/our-promise
Algae strains	Raceways	USA	https://www.microbioengineering.com/
	On-shore, off-shore, and modular cultivation technology options	USA	https://www.algaesystems.com/
Naturally occurring colonised micro-algae		Italy	https://www.algaewwt.com/
	Patented algae wheel technology	USA	https://www.algaewheel.com/
Algae blooms		USA	https://algix.com/
Different microalgae species		Brazil	https://www.algae.com.br/site/pt/
	Patented photobioreactors	USA	https://www.algamoil.com/
	Photobioreactors	Finland	https://www.redono.fi/
	Photobioreactors	USA	https://www.t2energy.com/
	Raceways	USA	https://www.eldoradobiofuels.com
	Raceways	Israel	https://www.aquanos.net
Assemblage of multi-species microalgae	Lagoons	USA	https://www.bio2solution.com/
	Raceways	Spain	https://www.all-gas.eu/contact-us
Consortia with a predominance of *Desmodesmus* sp. and *Scenedesmus obliquus* species	Raceway	Mexico	https://innovacion.iingen.unam.mx/AGEC-Microalgas.html

Preceding reviews discuss the potential of microalgae growth using wastewater, solely comparing biomass productivities under different types of wastewaters, cultivation systems, nutrient removals, and harvesting technologies (Gupta et al. 2019; Li et al. 2019; SundarRajan et al. 2019; Lu et al. 2020). Those reviews are not specifically directed towards looking into open outdoor reactors, long-term cultivation, and large-scale HRAP systems (> 0.4 m^3^). Published studies evaluating long-term cultivation of microalgae in large wastewater systems and under outdoor conditions report inconsistent environmental and operational factors (Chist 2016; Novoveská et al. 2016; Zhang et al. 2016). Therefore, it is often difficult to enable quantifiable comparisons. Recently, Nordio et al. (2023) highlighted temperature and irradiance as factors driving biomass productivity—N, P, and COD removals in two open-air pilot-scale HRAP reactors. They highlighted the need to analyse long-term nutrient recovery capacity and biomass productivity using models that include more parameters. This study conducts a profound systematic analysis of current global wastewater pilot-scale HRAP endeavours to identify key conditions needed for successful large-scale cultivations, detect key parameters, and highlight hotspots for process improvement. A meta-analysis was performed considering the following parameters: ammonia nitrogen (NH_4_^+^), nitrates (NO_3_^−^), orthophosphates (PO_4_^3−^), chemical oxygen demand (COD), pH, temperature (T°C), hydraulic retention time (HRT), solar radiation and biomass yield, to enable the discussion of environmental and process parameters that improve HRAP performance.

## Meta-analysis methodology

A multivariate analysis was carried out with the data obtained from the reviewed articles that met the inclusion criteria. Evaluated articles (Table 2) were statistically analysed for screening key parameters correlated to biomass productivity. Correlation and multiple regression analyses were performed to detect correlations between nutrients, environmental and operational parameters, and biomass production (*p* < 0.05). All statistical tests were performed with PAST version 4.03, STATGRAPHICS Centurion XVI.I and Minitab 19. The databases used for the search were Elsevier Scopus, ScienceDirect, and Google Scholar where scientific papers were located to ensure access to reliable data. The keywords used were “microalgae wastewater outdoor”, “microalgae wastewater cultivation”, “microalgae wastewater pond”, “wastewater algae raceway”, “microalgae large-scale”, and “microalgae pilot scale”. The databases and the keywords used were only the beginning of the research work; as the work progressed, the spectrum of databases and keywords expanded. Thus, other databases such as PubMed, Web of Science, and other general databases were also consulted. The selected articles were analysed in-depth to determine which ones met the following inclusion criteria: (1) microalgae cultivation in wastewater; (2) cultivation at large-scale (> 0.4 m^3^); (3) outdoor cultivation and raceways; (4) long-term cultivation (> 2 months); (5) articles that contained data about nutrients (NH_4_^+^, NO_3_^−^, PO_4_^3−^, and COD) and microalgae biomass yield; (6) studies with specifications regarding operational and environmental parameters (pH, T °C, HRT, and solar radiation). After a detailed examination of each article, the relevant data, according to the inclusion criteria previously established, were extracted to carry out a meta-analysis. Obtaining information under a systematic framework allowed the standardisation of reported values.

**Table 2:** Parameters reported for long-term microalgae pilot-scale cultivation using wastewater

Microorganisms	System/culture medium	Biomass yield (g L^−1^)	NH_4_^+^, NO_3_^−^, PO_4_^3−^ (mg L^−1^)	pH/HRT (days)	Temperature (°C)/solar radiation (W m^−2^)	COD (mg L^−1^)	BOD (mg L^−1^)/CO_2_	References
Microalgae/bacteria consortia *Chlamydomonas* spp. and *Microspora* dominant	Outdoor HRAP (0.464 m^3^) Piggery wastewater treated diluted 20- and 10-folds Primary treatment (rotatory screen, sedimentation)	0.33 to 0.38	NH_4_^+^ 33 to 60 NO_3_^−^ 3.5 PO_4_^3−^ N.D	8.45 / 10	7 / 185.33	536 to 949	N.R	De Godos et al. (2009)
Microalgae/bacteria consortia *Chlamydomonas* spp. and *Microspora* dominant	Outdoor HRAP (0.464 m^3^) Piggery wastewater treated diluted 20- and 10-folds Primary treatment (rotatory screen, sedimentation)	1.035 to 1.2	NH_4_^+^ 112 to 214 NO_3_^−^ 3 PO_4_^3−^ N.D	8.4 / 10	17 / 294.25	1221 to 2418	N.R	De Godos et al. (2009)
Microalgae/bacteria consortia *Chlamydomonas* spp. and *Microspora* dominant	Outdoor HRAP (0.464 m^3^) Piggery wastewater treated diluted 20- and 10-folds Primary treatment (rotatory screen, sedimentation)	1.17 to 1.33	NH_4_^+^133 to 264 NO_3_^−^ 0 PO_4_^3−^ N.D	8.35 / 10	15 /232.5	2316 to 4346	N.R	De Godos et al. (2009)
Consortium of 15 native algal isolates *Scenedesmus*, species dominant	Raceway Ponds (0.95 m^3^) carpet industry wastewater with sewage mix	1.47 g L^−1^	NH_4_^+^ 3.61 NO_3_^−^ 253.91 PO_4_^3−^ 21.95	7 / 9 (12)	15 and 25/N.A	106 to 183	331–487 (5–6%)	Chinnasamy et al. (2010)
*Pediastrum*, *Desmodesmus*, and *Micractinium*	Outdoor HRAP (8 m^3^) Anaerobic digester effluent	0.37 to 0.6	NH_4_^+^ 39 NO_3_^−^ N.D PO_4_^3−^ 5.4	7.1 / 4 and 8	21 / 227.55	110.2	272.8 (2 L min^−1^)	Park et al. (2010)
*Scenedesmus obliquus*	HRAP (0.53 m^3^) secondarilypretreatedwastewater (activated sludge, secondary sedimentation)	0.157	TN 26.1 TP 1.77	8.2 / 10	11 / 110.8	76.63	N.R	Arbib et al. (2013)
*Scenedesmus obliquus*	HRAP (0.53 m^3^) secondarily pretreated wastewater (activated sludge, secondary sedimentation)	0.190	TN 25 TP 2.23	8.7 / 10	11 / 135.9	83.33	N.R	Arbib et al. (2013)
*Scenedesmus obliquus*	HRAP (0.53 m^3^) secondarily pretreated wastewater (activated sludge, secondary sedimentation)	0.195	TN 24.92 TP 2.1	8.7 / 10	9 / 182.64	81.17	N.R	Arbib et al. (2013)
*Scenedesmus obliquus*	HRAP (0.53 m^3^) secondarily pretreated wastewater (activated sludge, secondary sedimentation)	0.245	TN 26.68 TP 2.03	9.3 / 8	18 / 227.33	77.36	N.R	Arbib et al. (2013)
Dominated 90% *Mucidosphaerium* *pulchellum*	HRAP (8 m^3^) domestic wastewater—primary influent to represent high nutrient load	0.23	NH_4_^+^ 39.70 NO_3_^−^ N.A PO_4_^3−^ 6.30	7.5 / 4	19 /31.8 to 488.9	N. A	N.R. (1%)	Sutherland et al. (2014)
Dominated 90% *Mucidosphaerium* *pulchellum*	HRAP (8 m^3^) domestic wastewater -50% primary influent: 50% tap water, to represent moderate nutrient load	0.18	NH_4_^+^ 19.90 NO_3_^−^ N.A PO_4_^3−^ 3.20	7.4 / 4	19 /31.8 to 488.9	N.A	N.R. (1%)	Sutherland et al. (2014)
Dominant microalgae (*Ulothrix* sp. or *Klebsormidium* sp.)	Outdoor raceway pond (12 m^3^) aquaculture wastewater	0.10	NH_4_^+^ 5 NO_3_^−^ 30 PO_4_^3−^ 1.50	9.78/ 8	20 / N.A	50	100–200 (5%)	Van Den Hende et al. (2016)
Warm season *Stigeoclonium* sp.; *Chlorella* sp.; *Monoraphidium*. Cold season *Chlorella* sp.; *Stigeoclonium* sp.	HRAP with two parallel lines each 1.5 m^2^ and a volume of 0.5 m^3^. real urban wastewater	Summer: 0.32 Winter: 0.11	Summer NH_4_^+^ 81 mg L^−1^ NO_3_^−^ N.A PO_4_^3−^ N.A Winter NH_4_^+^ 19 mg L^−1^ NO_3_^−^ N.A PO_4_^3−^ N.A	8/ 4	Summer: 25 / 282 Winter: 13 / 74	Summer: 52 ± 9 Winter: 67 ± 7	N.R	Matamoros et al. (2015)
Warm season *Stigeoclonium* sp.; *Chlorella* sp.; *Monoraphidium*. Cold season *Chlorella* sp.; *Stigeoclonium* sp.	HRAP with two parallel lines each 1.5 m^2^ and a volume of 0.5 m^3^. real urban wastewater	Summer: 0.35 Winter: 0.15	Summer NH_4_^+^ 81 mg L^−1^ NO_3_^−^ N.A PO_4_^3−^ N.A Winter NH_4_^+^ 19 mg L^−1^ NO_3_^−^ N.A PO_4_^3−^ N.A	9/ 8	Summer: 25 / 282 Winter: 13/ 74	Summer: 52 ± 12 Winter: 52 ± 7	N.R	Matamoros et al. (2015)
*Scenedesmus*	Three outdoor raceway (0.7, 0.8, 0.85 m^3^) primary domestic wastewater	0.457	NH_4_^+^ 63 NO_3_^−^ 1 PO_4_^3−^ 9	8 / 2.7	23 / 468	575	N.R. (20 L min^−1^)	Posadas et al. (2015)
*Scenedesmus*	Three outdoor raceway (0.7, 0.8, 0.85 m^3^) primary domestic wastewater	0.420	NH_4_^+^ 50 NO_3_^−^ 1 PO_4_^3−^ 11	8 / 2.8	22 / 462	744	N.R. (20 L min^−1^)	Posadas et al. (2015)
*Scenedesmus*	Three outdoor raceway (0.7, 0.8, 0.85 m^3^) primary domestic wastewater	0.408	NH_4_^+^ 74 NO_3_^−^ 0 PO_4_^3−^ 10	8/ 6.7	14 / 306	649	N.R. (20 L min^−1^)	Posadas et al. (2015)
*Scenedesmus*	Three outdoor raceway (0.7, 0.8, 0.85 m^3^) primary domestic wastewater	0.356	NH_4_^+^ 66 NO_3_^−^ 0 PO_4_^3−^ 9	8 / 6	13 / 300	432	N.R. (20 L min^−1^)	Posadas et al. (2015)
Consortium dominated by *Chlorella* sp.	Two outdoor HRAPs (0.47 m^3^). Raw urban wastewater; primary settler effluent	0.36	NH_4_^+^ 30	N.A. / 8	15.8 / 398	381	N.R	Gutierrez et al. (2016)
Consortium dominated by *Chlorella* sp.	Two outdoor HRAPs (0.47 m^3^). Raw urban wastewater; primary settler effluent	0.38	NH_4_^+^ 33	N.A. / 4	22.5 /446	463	N.R	Gutierrez et al. (2016)
Consortium dominated by *Chlorella* sp.	Two outdoor HRAPs (0.47 m^3^). Raw urban wastewater; primary settler effluent	0.46	NH_4_^+^ 36	N.A. / 6	23.7 /355	318	N.R	Gutierrez et al. (2016)
Consortium dominated by *Chlorella* sp.	Two outdoor HRAPs (0.47 m^3^). Raw urban wastewater; primary settler effluent	0.3	NH_4_^+^ 26	N.A. / 8	13.1 / 234	363	N.R	Gutierrez et al. (2016)
*Ulothrix* sp. and *Klebsormidium* sp.	Outdoor pilot-scale MaB-floc (10.5 m^3^). UASB and CAS effluent from food-industry	0.68	NH_4_^+^ 84.3 NO_3_^−^ 0.19 TP 14.79	8.6 / 35	12 / 89.9	635	6 a 318 (89 g/NM3, 5 − 8 L min^−1^)	Van den Hende et al. (2016)
*Scenedemus obliquus*	HRAP (0.53 m^3^) 1.93 m^3^ CAS effluent of WWTP	0.485	TN 20.46 TP 2.14	8 / 5	30.5 / 458	N.A	N.R. (CO_2_ pH 8 adjustment)	Arbib et al. (2017)
Mixed algal population (dominance of *Dictyosphaerium*)	Open HRAP (0.65 m^3^) Municipal WWTP influent partially settled	0.22	N 49.7 TP 2.4	8.3 / 7	18 / 90.28	N.A	N.R. (3 L min^−1^; 9%)	Gentili and Fick (2017)
*Pediastrum* sp.; *Chlorella* sp.; *Scenedesmus* sp.; and the cyanobacteria *Gloeothece* sp.	Hybrid HRAP (8.5 m^3^) Agricultural runoff	0.38	NH_4_^+^ 33.0 NO_3_^−^ 3 PO_4_^3−^ 1	8.3 / 10	7 / 185.33	526	N.R	García-Galán et al. (2018)
*Pediastrum* sp.; *Chlorella* sp.; *Scenedesmus* sp.; and the cyanobacteria *Gloeothece* sp.	Hybrid HRAP (8.5 m^3^) Agricultural runoff	1.206 (0.33)	NH_4_^+^ 60 NO_3_^−^ 4 PO_4_^3−^ 1	9.7 / 10	7/ 185.33	949	N.R	García-Galán et al. (2018)
Mixed culture of oleaginous yeast *Lipomyces starkeyi* and native microalgae (mostly *Scenedesmus* sp. and *Chlorella* sp.)	Raceway pond operating outdoors (0.40 m^3^) Raw urban wastewater (WWTP influent)	0.30	NH_4_^+^ 9 NO_3_^−^ N.R PO_4_^3−^ 12	11 / 14	25 ± 2 / 108.73	450	N.R	Iasimone et al. (2018)
*Scenedesmus* sp.; *Dictyosphaerium* sp.; *Chlamydomonas* sp.; *Euglena* sp.; *Micractinium* sp.; *Golenkinia*; Oocystis	Two HRAPs (total volume of 0.85 m^3^) WWTP lagoon system effluent (effluent from secondary lagoon 3)	0.092	NH_4_^+^ 19.7 NO_3_^−^ 2.9 TP 7.9	8.8 / 7	11.06 / 86.5	N.A	N.R	Wrede et al. (2018)
*Scenedesmus* sp.; *Dictyosphaerium* sp.; *Chlamydomonas* sp.; *Euglena* sp.; *Micractinium* sp.; *Golenkinia*,; *Oocystis*	Two HRAPs (total volume of 0.85 m^3^) WWTP Lagoon System Effluent (effluent from secondary lagoon 3)	0.029	NH_4_^+^ 25.5 NO_3_^−^ 8.7 TP 10.7	8.3 / 7	7.6 / 78.9	N.A	N.R	Wrede et al. (2018)
*Scenedesmus* sp.; *Dictyosphaerium* sp.; *Chlamydomonas* sp.; *Euglena* sp.; *Micractinium* sp.; *Golenkinia*; *Oocystis*	Two HRAPs (total volume of 0.85 m^3^) WWTP Lagoon System Effluent (effluent from secondary lagoon 3)	0.05	NH_4_^+^ 23.2 NO_3_^−^ 6.8 TP 12.3	8.5 / 7	13.36 / 145.6	N.A	N.R	Wrede et al. (2018)
*Scenedesmus* sp.; *Dictyosphaerium* sp.; *Chlamydomonas* sp.; *Euglena* sp.; *Micractinium* sp.; *Golenkinia*; *Oocystis*	Two HRAPs (total volume of 0.85 m^3^) WWTP Lagoon System Effluent (effluent from secondary lagoon 3)	0.129	NH_4_^+^ 2.6 NO_3_^−^ 4 TP 8.7	9.1 / 4	19.62 / 267.8	N.A	N.R	Wrede et al. (2018)
Consortium dominated by *Chlorella* sp.	Outdoor HRAP (0.47 m^3^) Municipal wastewater pre-treated (screening)	0.26 to 0.37	NH_4_^+^ 25 NO_3_^−^ 0.4 PO_4_^3−^ 2.3	8 / 4.5	17 /185.19	353 to 464	N.R	Arashiro et al. (2019)
Consortium dominated by *Chlorophyte*	Outdoor HRAP (1.9 m^3^) Pre-treated wastewater (after screening and removing grit, sand, and grease) from urban WWTP	0.19	NH_4_^+^ 98 NO_3_^−^ 1.3 PO_4_^3−^ 4.5	7.6 / 6	20 / 206.20	528	N.R	Galès et al. (2019)
Consortium dominated by *Chlorophyte*	Outdoor HRAP (1.9 m^3^) Pre-treated wastewater (after screening and removing grit, sand, and grease) from urban WWTP	0.26	NH_4_^+^ 91 NO_3_^−^ 0.5 PO_4_^3−^ 4.8	7.9 / 6	22 / 220.25	375	N.R	Galès et al. (2019)
Microalgae-bacteria consortia (*Chlorella* spp., *Scenedesmus* spp., and *Chlamydomonas* spp.)	Outdoor raceway reactor (1.2 m^3^) Supernatant from blackwater dewatering (centrate) of urban WWTP	0. 49	NH_4_^+^ 244 NO_3_^−^ 0.2 PO_4_^3−^ 5.7	8.2 / 10	25 / 320	112	N.R	Mantovani et al. (2020)
Microalgae-bacteria consortia *Chlorella* sp.; *Scenedesmus* sp.; *Chlamydomonas*	Raceway pond (1.2 m^3^) Supernatant from blackwater dewatering (centrate) of urban WWTP	0.23	NH_4_^+^ 244 NO_3_^−^ 0.2 PO_4_^3−^ 5.7	8.2 / 9	21/275	78–146	N.R	Mantovani et al (2020)
Natural selection of predominant species	Pilot-scale HRAP (working volume of 22 m^3^) synthetic urban wastewater	0.2	NH_4_^+^ 17.3 NO_3_^−^ N.R PO_4_^3−^ 3.9	7.3–9 / 6	22 / 94.13	277	N.R	Robles et al. (2020)
Microalgal community dominated by *Ankistrodesmus falcatus* (Corda) Ralfs (winter, spring) and *Micractinium pusillum* Fresenius (summer)	Outdoor HRAP (1.5 m^3^)	0.131	NH_4_^+^ 38.2 NO_3_^−^ 0.9 PO_4_^3−^ 6.6	7.5 / 8	8.9 /85.04	165.3	N.R	Sutherland et al. (2020a)
Microalgal community dominated by *Ankistrodesmus falcatus* (Corda) Ralfs (winter, spring) and *Micractinium pusillum* Fresenius (summer)	Outdoor HRAP (90 m^3^)	0.124	NH_4_^+^ 38.2 NO_3_^−^ 0.9 PO_4_^3−^ 6.6	7.6 / 8	11.1 /85.04	160.95	N.R	Sutherland et al. (2020a)
Microalgal community dominated by *Ankistrodesmus falcatus* (Corda) Ralfs (winter, spring) and *Micractinium pusillum* Fresenius (summer)	Outdoor HRAP (2900 m^3^)	0.107	NH_4_^+^ 38.2 NO_3_^−^ 0.9 PO_4_^3−^ 6.6	7.3 / 8	11 /85.04	139.2	N.R	Sutherland et al. (2020a)
Microalgal community dominated by *Ankistrodesmus falcatus* (Corda) Ralfs (winter, spring) and *Micractinium pusillum* Fresenius (summer)	Outdoor HRAP (1.5 m^3^)	0.147	NH_4_^+^ 35.3 NO_3_^−^ 1.1 PO_4_^3−^ 5	7.7 / 8	21.1 / 187.39	174	N.R	Sutherland et al. (2020a)
Microalgal community dominated by *Ankistrodesmus falcatus* (Corda) Ralfs (winter, spring) and *Micractinium pusillum* Fresenius (summer)	Outdoor HRAP (90 m^3^)	0.144	NH_4_^+^ 35.3 NO_3_^−^ 1.1 PO_4_^3−^ 5	7.9 / 8	19.3 / 187.39	187	N.R	Sutherland et al. (2020a)
Microalgal community dominated by *Ankistrodesmus falcatus* (Corda) Ralfs (winter, spring) and *Micractinium pusillum* Fresenius (summer)	Outdoor HRAP (2900 m^3^)	0.119	NH_4_^+^ 35.3 NO_3_^−^ 1.1 PO_4_^3−^ 5	7.5 / 8	19.3 / 187 .39	153.7	N.R	Sutherland et al. (2020a)
Microalgal community dominated by *Ankistrodesmus falcatus* (Corda) Ralfs (winter, spring) and *Micractinium pusillum* Fresenius (summer)	Outdoor HRAP (1.5 m^3^)	0.211	NH_4_^+^ 56.8 NO_3_^−^ 0.9 PO_4_^3−^ 5.5	8.7 / 8	25.1 / 267.06	276.95	N.R	Sutherland et al. (2020a)
Microalgal community dominated by *Ankistrodesmus falcatus* (Corda) Ralfs (winter, spring) and *Micractinium pusillum* Fresenius (summer)	Outdoor HRAP (90 m^3^)	0.237	NH_4_^+^ 56.8 NO_3_^−^ 0.9 PO_4_^3−^ 5.5	8.7 / 8	22.9 / 267.06	311.75	N.R	Sutherland et al. (2020a)
Microalgal community dominated by *Ankistrodesmus falcatus* (Corda) Ralfs (winter, spring) and *Micractinium pusillum* Fresenius (summer)	Outdoor HRAP (2900 m^3^)	0.168	NH_4_^+^ 56.8 NO_3_^−^ 0.9 PO_4_^3−^ 5.5	8.1 / 8	23 / 267.06	220.4	N.R	Sutherland et al. (2020a)
*Coelastrum microporum*; *Micractinium pusillum*; *Microcystis aeruginosa*	HRAP 2900 m^3^/digested effluent of the covered anaerobic pond	0.224	NH_4_^+^ 37.3 NO_3_^−^ 0.7 PO_4_^3−^ 5	9.3 / 8	20.7 / 155.09	107.59	N.R	Sutherland et al. (2020b)
*Cyanobacterium Microcystis aeruginosa* *Desmodesmus opoliensis*	HRAP 2900 m^3^/digested effluent of the covered anaerobic pond	0.168	NH_4_^+^ 23 to 40.6 NO_3_^−^ 1.1 PO_4_^3−^ 3.2 to 5.6	8.9 / 4	21.2 / 152.78	84	N.R	Sutherland et al. (2020b)
*Chlorella* sp. *Scenedesmus* sp. (72%/22%)	Semi-closed HRAP (2m^3^) Digestate from anaerobic digestion of dairy wastewater	1.80	NH^4+^ 200 NO_3_^−^ 0 PO_4_^3−^ 40	7.3 / 26	22/500	799–813	380–492	Debowski et al. (2020)
*Scenedesmus* sp.	Pilot-scale raceway photobioreactors (11.3 m^3^) University domestic wastewater with solid removal Winter—dilution rates (0.1 day^−1^)	0.45	NH^4+^ 180 NO_3_^−^ 5 PO_4_^3−^ 12	8.0 ± 1.0 /10	16 / 118.61	N.A	N.R	Morillas-España et al. (2021)
*Scenedesmus* sp.	Pilot-scale raceway photobioreactors (11.3 m^3^) University domestic wastewater with solid removal Winter—dilution rates (0.2 day^−1^)	0.45	NH^4+^ 180 NO_3_^−^ 5 PO_4_^3−^ 12	8.0 ± 1.0 / 5	16 / 118.61	N.A	N.R	Morillas-España et al. (2021)
*Scenedesmus* sp.	Pilot-scale raceway photobioreactors (11.3 m^3^) University domestic wastewater with solid removal Winter—dilution rates (0.3 day^−1^)	0.25	NH^4+^ 170 NO_3_^−^ 5 PO_4_^3−^ 8	8.0 ± 1.0 / 3	20 / 166.06	N.A	N.R	Morillas-España et al. (2021)
*Scenedesmus* sp.	Pilot-scale raceway photobioreactors (11.3 m^3^) University domestic wastewater with solid removal Spring—dilution rates (0.1 day^−1^)	0.50	NH^4+^ 200 NO_3_^−^ 3 PO_4_^3−^ 9	8.0 / 10	22 / 237.22	N.A	N.R	Morillas-España et al. (2021)
*Scenedesmus* sp.	Pilot-scale raceway photobioreactors (11.3 m^3^) University domestic wastewater with solid removal Spring—dilution rates (0.2 day^−1^)	0.60	NH^4+^ 200 NO_3_^−^ 3 PO_4_^3−^ 9	8.0 ± 1.0 /5	22 / 237.22	N.A	N.R	Morillas-España et al. (2021)
*Scenedesmus* sp.	Pilot-scale raceway photobioreactors (11.3 m^3^) University domestic wastewater with solid removal Spring—dilution rates (0.3 day^−1^)	0.25	NH^4+^ 200 NO_3_^−^ 3 PO_4_^3−^ 9	8.0 ± 1.0 / 3	22 / 237.22	N.A	N.R	Morillas-España et al. (2021)
*Scenedesmus* sp.	Pilot-scale raceway photobioreactors (11.3 m^3^) University domestic wastewater with solid removal Summer—dilution rates (0.1 day^−1^)	0.60	NH_4_^+^ 210 NO_3_^−^ 1 PO_4_^3−^ 12	8.0 ± 1.0 / 10	26/ 284.67	N.A	N.R	Morillas-España et al. (2021)
*Scenedesmus* sp.	Pilot-scale raceway photobioreactors (11.3 m^3^) University domestic wastewater with solid removal Summer—dilution rates (0.2 day^−1^)	0.80	NH_4_^+^ 210 NO_3_^−^ 1 PO_4_^3−^ 12	8.0 ± 1.0 / 5	26/ 284.67	N.A	N.R	Morillas-España et al. (2021)
*Scenedesmus* sp.	Pilot-scale raceway photobioreactors (11.3 m^3^) University domestic wastewater with solid removal Summer—dilution rates (0.3 day^−1^)	0.30	NH_4_^+^ 210 NO_3_^−^ 1 PO_4_^3−^ 12	8.0 ± 1.0 /3	26/ 284.67	N.A	N.R	Morillas-España et al. (2021)
*Scenedesmus* sp.	Pilot-scale raceway photobioreactors (11.3 m^3^) University domestic wastewater with solid removal Autumn—dilution rates (0.1 day^−1^)	0.50	NH_4_^+^ 170 NO_3_^−^ 5 PO_4_^3−^ 8	8.0 ± 1.0 / 10	20 / 166.06	N.A	N.R	Morillas-España et al. (2021)
*Scenedesmus* sp.	Pilot-scale raceway photobioreactors (11.3 m^3^) University domestic wastewater with solid removal. University domestic wastewater with solid removal Autumn—dilution rates (0.2 day^−1^)	0.65	NH_4_^+^ 170 NO_3_^−^ 5 PO_4_^3−^ 8	8.0 ± 1.0/5	20 / 166.06	N.A	N.R	Morillas-España et al. (2021)
*Scenedesmus* sp.	Pilot-scale raceway photobioreactors (11.3 m^3^) University domestic wastewater with solid removal Autumn—dilution rates (0.3 day^−1^)	0.25	NH_4_^+^ 170 NO_3_^−^ 5 PO_4_^3−^ 8	8.0 ± 1.0/3	20 / 166.06	N.A	N.R	Morillas-España et al. (2021)

*N.D.*, not detected; *N.A.*, not available; *N.R.*, not reported; *TKN*, total Kjeldahl nitrogen; *NH*_*4*_, ammonium; *N*, nitrogen; *P*, phosphorous; *TN*, total nitrogen; *TP*, total phosphorous; *HRT*, hydraulic retention time

The meta-analysis helped identify key parameters that have the highest impact on biomass production and hotspots. As most of the selected studies reported different units related to solar radiation; to standardise the data, it was decided to obtain this information from NASA Power Data Access Viewer for every location. In the meta-analysis study, reactors operating at ambient conditions and natural solar radiation were considered. The different units were unified according to the international system of units and considering the dimensions of the high-rate algal ponds, respectively. Photosynthetically active radiation (PAR) is a term used to describe radiation in wavelengths useful for photosynthesis of plants. PAR is generally accepted to be wavelengths between 400 a 700 nm. Solar radiation is usually reported in units of μmol m^−2^ s^−1^ PAR, W m^−2^ Μmol m^−2^ s^−1^ is an SI unit, and the approximation 1 W m^−2^ ≈ 4.6 μmol m^−2^ s^−1^ (Sager and McFarlane 1997). The parameter “all sky insolation incident on a horizontal surface” was used because it represents the monthly average amount of total solar radiation on the earth’s surface.

Daily biomass areal and volumetric productivities, plus specific growth rates, were calculated using Eqs. 1, 2, and 3, respectively. Specific growth rates refer to the rate of increase of biomass of a cell population per unit of biomass concentration.

1 $$\text{Areal Biomass Productivity}=\frac{ \left(\mathrm{Ct}-\mathrm{Co}\right){V}_{r}}{\mathrm{HRT}(\mathrm{Area})}$$

2 $$\mathrm{Volumetric}\text{ Biomass Productivities}=\text{Areal Biomass Productivity} \left(\frac{\mathrm{Area}}{\mathrm{Vr}}\right)$$

3 $$\mathrm{Sg}=\frac{\mathrm{ln}\;Ct-\mathrm{ln}\;Co}{t}$$

*Sg* stands by specific growth rate; *Ct* and *Co* are the biomass or cell densities at time *t*, and 0, respectively; *Vr* is the raceway pond volume, *HRT* is the hydraulic residence time, and the area is the raceway pond area.

A limitation of this meta-analysis is that the number of papers reporting pilot-scale work is not currently vast for scalable microalgae wastewater treatment systems using HRAP. However, most papers tested several conditions which increased the data input in the meta-analysis. Also, studies by De Godos et al. (2009) were not considered as they can bias biomass interpretation. De Godos et al. (2009) reported influent organic concentrations 4 times that of the effluent organic concentrations, so it is highly plausible that not all effluent biomass was algal/bacterial based. Private sector pilot projects (Table 1) were excluded due to a lack of systematic data.

## Results and discussion

Wastewater treatment using microalgae for nutrient removal has been studied for more than 50 years. The first studies reported by Caldwell (1946) and Oswald and Gotaas (1957) used microalgae for wastewater treatment in oxidation ponds, demonstrating that their large-scale cultivation could be used for wastewater treatment. The use of oxidation ponds became important to avoid wastewater discharges in freshwater bodies and coastal waters that can cause water eutrophication, leading to serious sanitary and ecological consequences. Since then, conventional wastewater treatment has moved away from microalgal proliferating oxidation ponds to bacterial systems. However, microalgal biomass produced from wastewater has the potential to reduce CO_2_ emissions, provide water treatment, and generate value-added products.

Table 2 shows 69 HRAP cultivation conditions gathered from 23 scientific articles retrieved from the literature that met the inclusion criteria for the analysis. To optimise yields and productivity in HRAP wastewater treatment, various factors merit consideration, including the careful selection of microalgae for inoculation and subsequent consortia formation. Adaptation to fluctuations in wastewater composition stands as a pivotal determinant. Outdoors, mixed microalgae-bacteria polycultures emerge as the foremost viable option for cultivation, typically characterised by the dominance of a particular microalgae genus. Geographical location, environmental conditions, and the physicochemical properties of the wastewater shape the selection or prevalence of microalgal taxa. Notably efficient genera for wastewater treatment include *Chlorella*, *Scenedesmus*, and *Chlamydomonas* (González-Camejo et al. 2021). The literature reviewed in Table 2 highlights *Scenedesmus* as the predominant taxon under outdoor conditions. A review by Dolganyuk et al. (2020), based on data from Nancucheo and Johnson’s data (2012), suggests the following: a temperature of 29 °C, a culture duration of 12 days, agitation at 90 rpm, and a pH of 7.1 as optimal culture conditions for *Scenedesmus obliquus*. However, there is a reported phenomenon wherein the initially dominant taxa can be supplanted by other genera or species as the process unfolds. This transition is particularly pronounced in consortia featuring a diverse array of microalgae. As such, the microalgal taxon was not a parameter included in the metadata analysis, but the information presented in this study provides a guide on the selection of species for outdoor pilot-scale wastewater treatment. The construction of a well-suited microalgae-bacteria consortium via meticulous species selection and establishing an appropriate microalgae-to-bacteria ratio becomes imperative for enhancing wastewater treatment performance. Remarkably, microalgae-bacteria cultures exhibit adaptability to a broad spectrum of environmental parameters, spanning pH, irradiance, temperature, and nutrient concentrations.

Table 2 also shows the change of parameters according to yearly variations. Morillas-España et al. (2021) present a study of reactors installed in Almeria, Spain, and report average and maximum solar radiation and temperature incidences for these seasons: 1200–2000 µmol m^−2^ s^−1^ and 26–34 °C. The highest nutrient concentrations (NH_4_^+^, PO_4_^3−^), as well as removal efficiencies and biomass yields, occur during the summer. Overall, the spring to summer seasons provide the most favourable conditions for cultivation, whilst the summer and autumn environmental parameters may require compensation based on location.

The existing literature describes different types of wastewaters that can be treated by microalgae cultivation at the laboratory or pilot scale. Table 3 shows a summary of the type of effluent used in the pilot-scale studies included in this meta-analysis, either combined or individually. The most studied type of wastewater is primarily settled municipal wastewater, followed by anaerobic digestion and treated wastewater. Treated wastewater will have lower concentration of COD than settled wastewater. Of note is the use of anaerobic digestion effluent (digestate) containing high concentrations of nutrients, in particular, rich in ammonia, resulting in a high yield of microalgae biomass as shown in Table 2.

**Table 3:** Analysis of pilot-scale studies revised according to the type of wastewater utilised

Wastewater used	Number of evaluated conditions
Agricultural runoff + municipal wastewater	3
Anaerobic digestion effluent	1
Aquaculture wastewater	1
Carpet mill wastewater (90%) and municipal wastewater (10%)	1
Conventional activated sludge effluent	1
Digestate from anaerobic digestion	3
Digested effluent from an anaerobic pond treating municipal wastewater	13
Diluted piggery wastewater	2
Municipal wastewater	5
Primary settled domestic wastewater (high nutrient load)	1
Primary settled domestic wastewater (moderate nutrient load)	1
Settled municipal wastewater	28
Treated wastewater	9
Grand total	69

### Microalgal physiology and biomass productivity in outdoor cultivation

Microalgal physiology in outdoor cultivation is an aspect that directly influences biomass productivity. During the photosynthetic process, microalgae can consume not only CO_2_, but also macro- and micronutrients from wastewater, which can result in high growth rates and therefore biomass productivity (Santos et al. 2019). This has been estimated to be up to 50 times higher than that of grasslands (Chisti 2007). Biomass productivity, particularly in outdoor cultivation, is directly influenced by cell physiology, which is a function of various conditions such as the components of the culture medium (e.g. type of wastewater), pH, temperature, growth phase (culture time), and an efficient harvesting method or even stirring of the culture (Dolganyuk et al. 2020). However, light is an overwhelming factor that has a direct impact on the photosynthetic activity of microalgae (Rincon et al. 2019). Maximum growth occurs during the day, but up to 25% of the biomass produced can be lost during the night through respiration, depending on the light intensity during daytime cultivation and the day and night temperature (Chisti 2007). The absence of light reduces the rate of photosynthesis and thus the conversion of solar energy into chemical energy compounds such as adenosine triphosphate (ATP) and nicotinamide adenine dinucleotide phosphate (NADPH) to support cell growth (Baba and Shiraiwa 2013).

Moreover, excessive light can damage the chlorophyll in microalgal cells. In this sense, microalgae can be classified into photoautotrophic, heterotrophic, and mixotrophic cultures. These are based on how they use light as an energy source for growth. Photoautotrophic: use sunlight as an energy source and CO_2_ as a carbon source. Heterotrophic: can grow under phototrophic conditions and can also make use of organic carbon in the absence of light. Mixotrophic: cell growth is based on CO_2_ and organic carbon, so both the photosynthetic and organic substrate pathways must function simultaneously (Li et al. 2016; Rincon et al. 2019; Dolganyuk et al. 2020). Microalgae cultivation in large-scale systems, mainly outdoor, is usually photoautotrophic (Dolganyuk et al. 2020), where the stability of the cultures and hence biomass productivity will be highly dependent on environmental conditions.

### Nutrient composition and removals

**Fig. 3:**
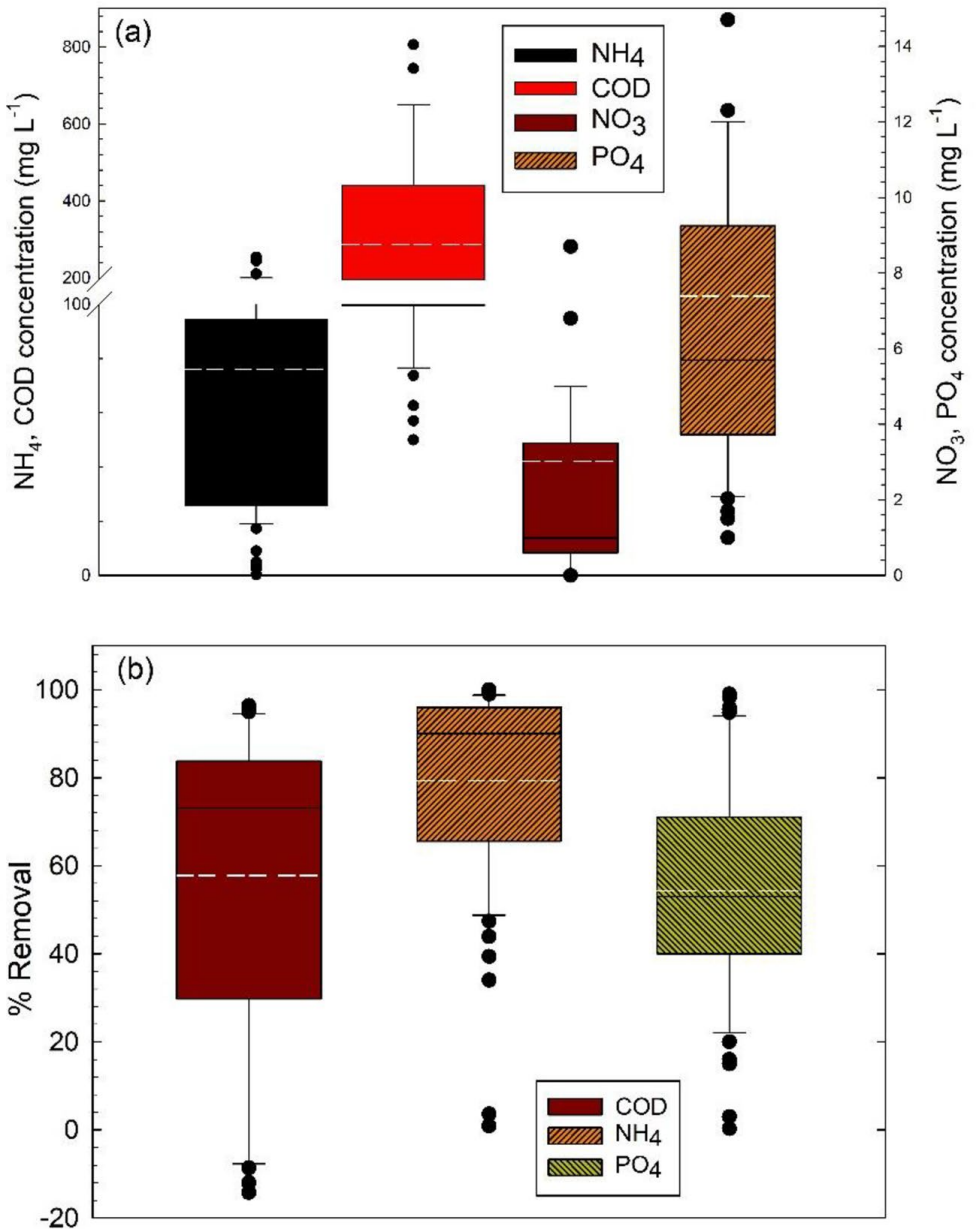
Box plots of the data collected and calculated from the literature in Table 2, for microalgal growth in wastewater in high-rate ponds. **a** Chemical oxygen demand and nutrient concentrations (mg L^−1^) and **b** removals (%). White lines denote the mean of values

The use of wastewater for its abundant amount of nutrients for microalgae growth and simultaneously as tertiary wastewater treatment has been widely investigated due to the high removal efficiencies achieved (Li et al. 2019). The use of wastewater saves the cost of nutrient supplementation and enhances the assimilation of organic and inorganic pollutants during microalgal cell metabolism. Nitrogen and phosphorus are the most important nutrients for microalgal growth. Microalgae utilise inorganic and organic nitrogen in the form of nitrate (NO_3_^−^), ammoniacal nitrogen (NH_4_^+^), and urea (CO(NH_2_)_2_). Ammoniacal nitrogen is the preferred source of nitrogen, whilst the form of free phosphorus assimilable by microalgae is orthophosphates (PO_4_^3−^) (Komolafe et al. 2014). The box plot in Fig. 3a show the concentrations of nitrate (NO_3_^−^), ammonia nitrogen (NH_4_^+^), and orthophosphate (PO_4_^3−^) as main nutrients for the growth of microalgae in HRAP.

Within the different types of wastewaters used, the highest microalgal biomass yields have been reported when using effluents with high organic loads such as leachate, centrate, and piggery wastewater. However, we can see that ammonia nitrogen loads used in most pilot scale studies are not excessively high, even nitrates, and phosphate concentrations are in the low range. Municipal wastewater effluent normally contains around 30 mg L^−1^ of ammonia nitrogen; from the data gathered here, wastewater concentrations of ammonia averaged 60 mg L^−1^, whilst nitrate and phosphate concentrations were ten times lower between 4 and 8 mg L^−1^. The variations in concentration with respect to the average ammonium values are attributed to the wastewater origin. Orthophosphates and nitrates are consistently present at low concentrations; the latter is considered a second source of nitrogen. Consequently, the removal percentages are relatively proportional to the requirements that microalgae have for each of them. This is related to the elementary composition of microalgae and the minimum requirements of basic nutrients for their growth. Based on the elementary composition of microalgae and from the conservation of mass, the minimal requirement for 1 g microalgal biomass (DW) production might be approximated as 1.83 g of CO_2_, 0.07 g N, and 0.008 g P (Beal et al. 2012; Romero et al. 2017). As such, to obtain a concentration of 1 g microalgae L^−1^ of wastewater requires a minimum of 70 mg L^−1^ of N and 8 mg L^−1^ P.

COD utilised is in the range of 150 to 450 mg L^−1^ which is a typical interval for municipal wastewater. Additionally, COD can be considered an indicator of the overall removal of contaminant matter in raceway-type wastewater lagoons. The variations with respect to the average % removal can be attributed to the complexity of the wastewater used. HRAP can effectively remove 90% of NH_4_^+^, 70% of COD, and 50% of PO_4_^3−^ from wastewater (Fig. 3b). The highest removals are obtained for ammonia, followed by COD, and finally phosphate.

### Effect of wastewater, operational, and environmental factors on biomass yield productivity

**Fig. 4:**
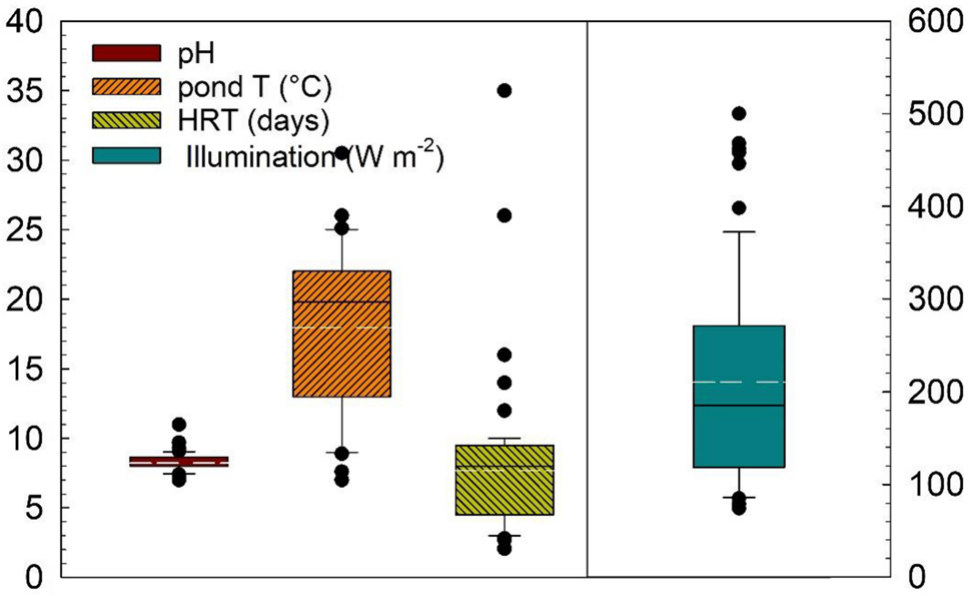
Box plots of irradiation, pH, HRT, and temperature conditions for pilot scale studies depicted in Table 2. All of the 69 evaluated conditions were included for analysis. White lines denote the mean of values. Illumination used the value of 4.57 to convert Watts m^−2^ to μmol m^−2^ s^−1^ (PAR, photosynthetic irradiance radiation) and vice versa

Figure 4 shows environmental conditions mainly used in pilot scale studies. Whilst microalgae irradiation has a mean of 210 W m^−2^ or 962 μmol m^−2^ s^−1^ which is a normal average for most countries in the world (Fig. 2). Open systems report an average HRT between 5 and 10 days (with a mean of 7.7, Table 2), a similar range is also observed for closed systems. Temperature utilised lies between 12 and 24 °C with a mean of 18 °C and pH between 8 and 9, with a mean of 8.2. The range of temperatures used corresponds to countries with daily total irradiations between 6 and 9 kWh m^−2^. An alkaline pH is expected as microalgae growth produces hydroxyl ions (Eze et al. 2018). The biomass productivity of the algae culture decreases with an excessive increase in pH of the medium, which causes ammonia volatilisation and a decreased carbon uptake. According to Eze et al. (2018), under optimal pH conditions for microalgae cultivation (8.1), a carbon fixation efficiency of about 99% can be achieved. The supply of CO_2_ from an external source can be used to control pH (8–9) and to compensate for carbon limitations. Studies using primary wastewater and effluents from secondary treatments for microalgae-based treatment have reported average C, N, and P ratios of 100:19:3 and 100:34:7, respectively (Mohsenpour et al. 2021). The intrinsic link between pH and carbon dioxide concentration indicates that on-line monitoring of parameters such as pH is a good option to control an ensure an optimum HRAP performance.

**Fig. 5:**
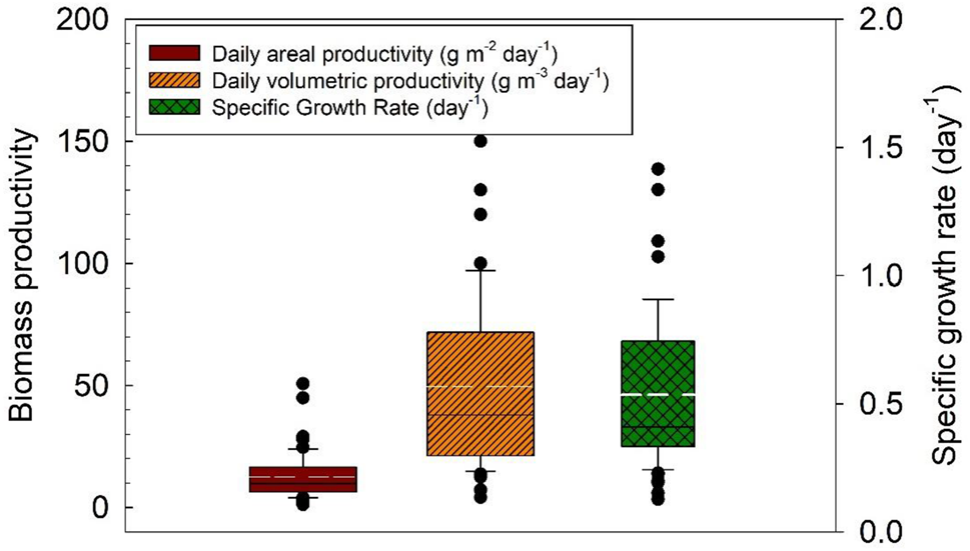
Biomass productivity and specific growth rates derived from pilot-scale raceway ponds using wastewater. All of the 69 evaluated conditions depicted in Table 2 were included for analysis. White lines denote the mean of values

Biomass yield productivities and calculated specific growth rates are depicted in Fig. 5. Volumetric productivity is approximately five times higher than the areal productivity, denoting how the extensive area needed for HRSP decreases areal productivity; reported ranges of microalgal biomass production coupled with wastewater treatment using HRAP vary widely from 5.7 to 40 g m^−2^ day^−1^ (Morales-Amaral et al. 2015; Robles et al. 2020; Sutherland et al. 2020a). The largest HRAP reported is a full-scale system using working volumes of 2900 m^3^ (Sutherland et al. 2020a). However, studies reporting > 0.4 m^3^ systems are scarce (Table 1). Most of the studies, at large-scale, have been performed at a pilot scale between 0.15 and 8 m^3^. The calculated specific growth rate has an average of ~ 0.54 day^−1^ which is half the value reported experimentally of ~ 1 day^−1^ in previous publications (Wang et al. 2010; Basu et al. 2015). This denotes a significant decrease in microalgae growth when comparing environmental systems to laboratory experiments. This should inform when modelling and scaling-up laboratory data for a larger system implementation. Studies performed on a laboratory scale cannot predict by themselves the performance and capacity of the different strains of microalgae under outdoor conditions where large fluctuations of solar irradiation, temperature, pH, and predation by other microorganisms would be present, and therefore, the species evaluation should be carried under such conditions. Wen et al. (2016) evaluated the scaling-up of *Graesiella* sp. first cultivated in 10 L reactors under artificial irradiation and subsequently scaled to 30 L reactors under natural conditions of solar irradiation. Changes in environmental conditions produced a decrease in lipid content (from 45 to 34%). García et al. (2017) cultivated *Chlorella vulgaris* in open photobioreactors using diluted swine wastewater. They compared outdoor and laboratory photobioreactors and observed that phosphorus and total organic carbon removal efficiencies were higher in laboratory conditions, whilst nitrogen removal was higher in outdoor photobioreactors; this removal was mainly attributed to NH_4_^+^ volatilisation, since the assimilation in the biomass was only 25 to 44% of the total nitrogen removed.

**Fig. 6:**
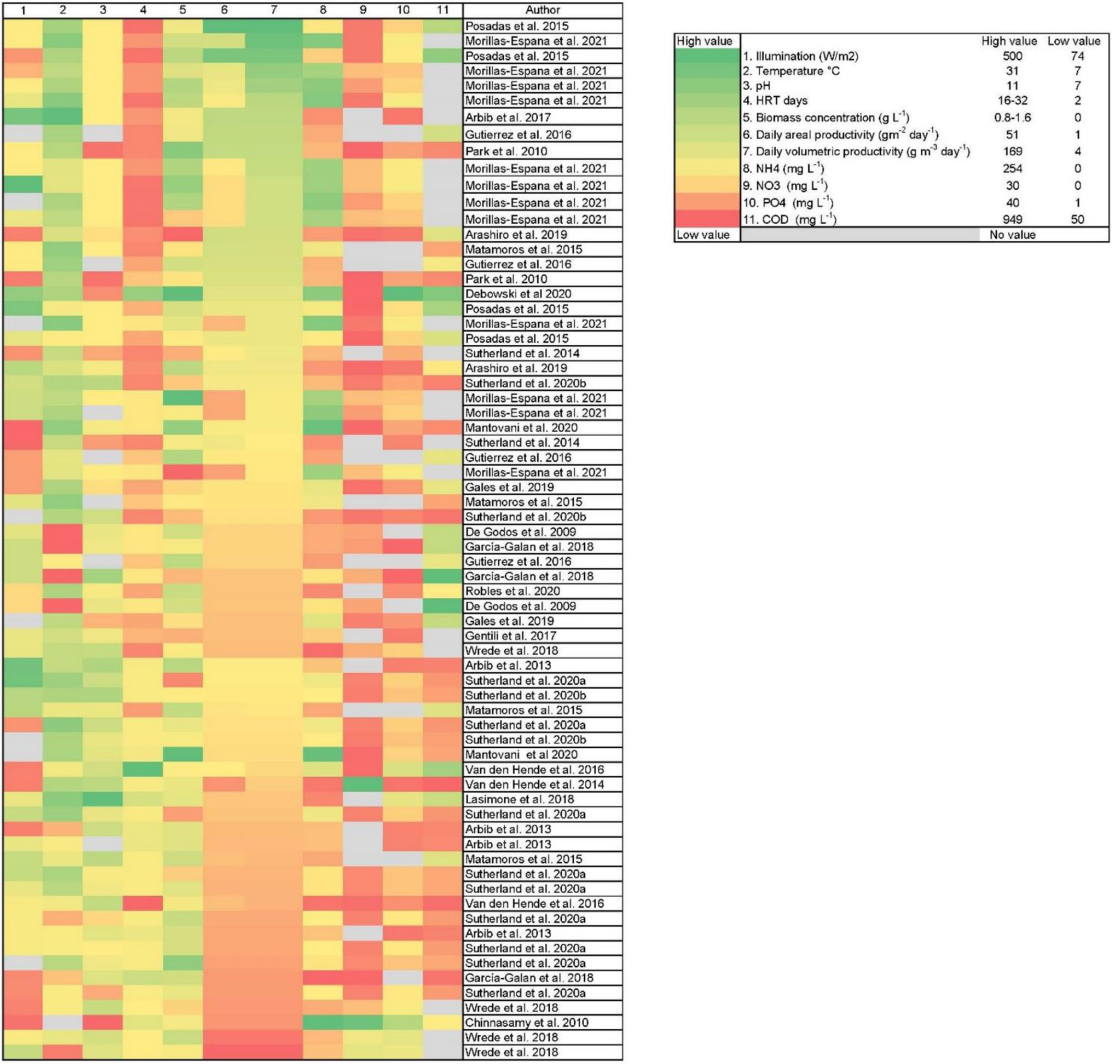
A heatmap combining the performance parameters of each pilot-scale microalgal study. All of the 69 evaluated conditions depicted in Table 2 were included. The colour grey denotes that data is absent. Gradients are scaled as a percentage of each parameter’s high (100%, green) or low values (0%, red) to compare them equally

Pilot-scale studies were mapped to visualise the correlation and prediction of the biomass yield with respect to growth conditions and wastewater concentrations (Fig. 6). Most studies reported multiple parameters per set-up, as such parameters tested are shown for the same authors. Microalgae exposure to environmental conditions has an impact on biomass production. It is observed that Morillas-España et al. (2021) and Posadas et al. (2015) recorded the highest productivities, as they used wastewater with low to medium–high ammonium concentrations and medium to highest irradiation. Both authors report similar daily volumetric productivities (120–169 g m^−3^ day^−1^); however, the highest daily areal production (50 g m^−2^ day^−1^ obtained by Posadas et al. (2015) stands out. This can be attributed to the small cultivation area used (raceway: 2.7 m^2^; 0.8 m^3^), where irradiation and mixing can be enhanced, compared to the one used by Morillas-España et al. (2021) (raceway: 80 m^2^; 11.8 m^3^).

**Fig. 7:**
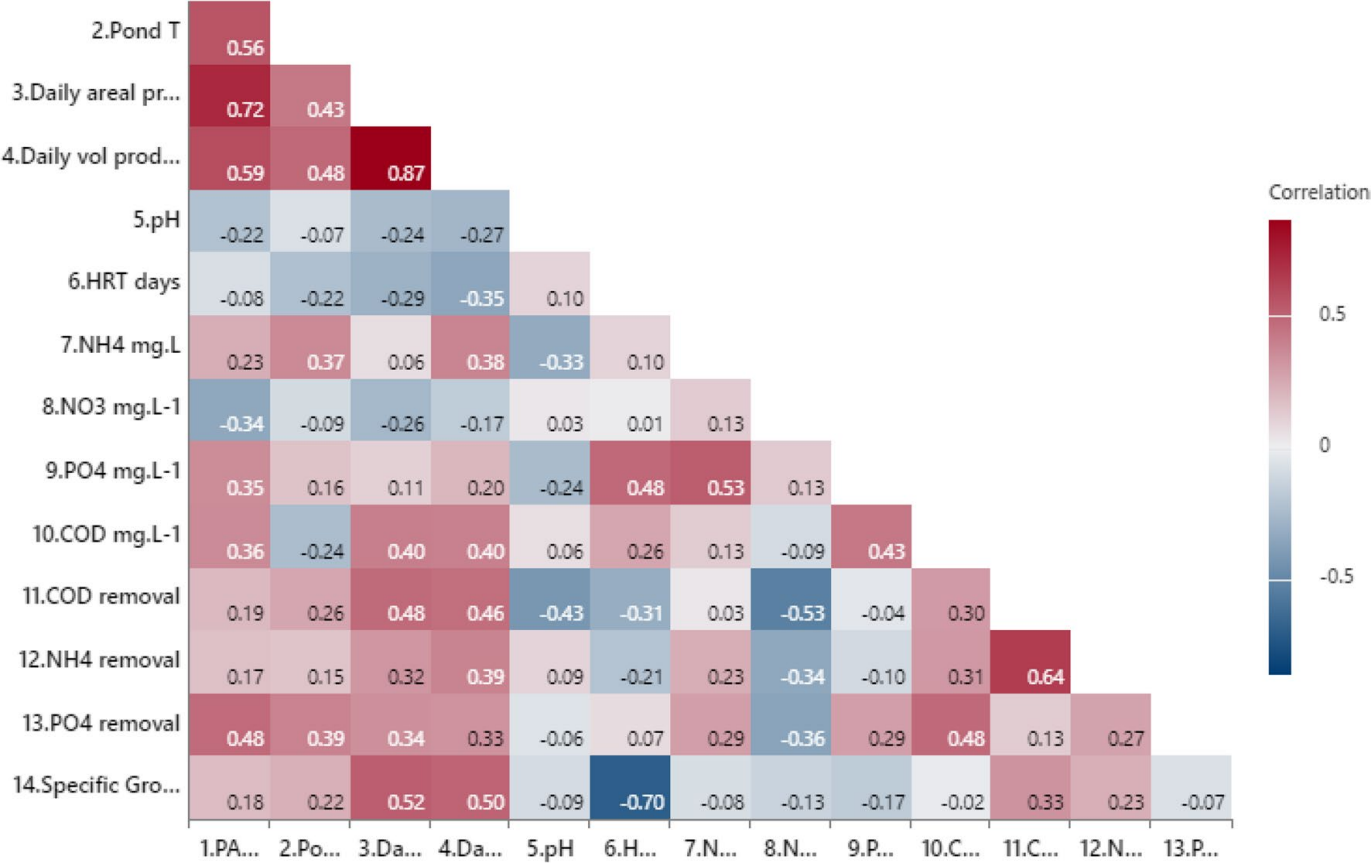
Correlogram between nutrients, environmental/operational parameters, and microalgae biomass productivity. The red colours indicate a positive correlation whilst blue colours indicate a negative correlation. Label 1 is the illumination factor as PAR, and 14 is the specific growth rate. Variables that have the darkest colours have the highest Pearson correlation coefficients and vice versa

Figure 7 displays a correlogram among 14 variables analysed. With respect to operational and environmental factors on the performance of microalgae grown in wastewater, it was observed that only ammonia concentrations had a positive significant effect on biomass production, whilst nitrate had a negative effect. The correlogram helped initiate investigations towards determining the relationship between variables. It can be seen that volumetric and areal productivities are highly correlated due to their dependency. Also, temperature and illumination plus COD with nutrient concentrations show a high correlation due to their dependant effect.

### Parameter prediction on biomass yield productivity

Statistical investigations on combined effects on productivity implied the removal of 12 insignificant or dependant variables. It was found that illumination (as PAR) and ammonia concentration were significant, with no convoluted variables that explained 80% of the calculated biomass productivity, whilst the range of changes in HRT had a less significant effect. According to the Pearson correlation, NH_4_^+^ and PAR have the greatest impact on biomass production. Multiple regression analyses indicated that 79.94% of the variance (*R*^2^) in the analysed dependent variable, biomass volumetric productivity, can be explained by NH_4_^+^ and PAR (Fig. 9). The importance of illumination highlights the need to consider the climatic characteristics of the HRAP location. An ideal illumination improves microalgae biomass productivity when cultivated outdoor and at large-scale. Solar radiation directly affects the photosynthetic activity of algae (Arbib et al. 2013) and their growth rate. This directly impacts the regulation of cell proliferation, biocomponent accumulation, and metabolic changes in microalgae, as light quality plays an important role (Hong et al. 2023). Microbial growth rate increases with increasing light intensity, and to verify this relationship, several models have been proposed (Xu et al. 2019). Solar irradiation can also affect CO_2_ fixation since according to Toro-Huertas et al. (2019), about 30% of the total CO_2_ fixed by microalgae was carried out at low irradiation. Environmental conditions change over time and not only affect growth but also cause fluctuations in the abundance of species (Newby et al. 2016). Table 2 shows studies performed in different seasons using the same reactor to evaluate how environmental conditions such as solar radiation and temperature influence the biomass production. For example, De Godos et al. (2009) evaluated the performance of two 464 L HRAP under different climatic conditions from January to October, and they found that the optimum environmental conditions were from July to September, producing the highest biomass yield of 1.33 g L^−1^. During this period, the temperature and solar radiation were higher compared to the January–June period. Following the same line, Sutherland et al. (2020a) assessed the performance of three different-size HRAPs over three seasons and reported the highest biomass yield during the summer.

**Fig. 8:**
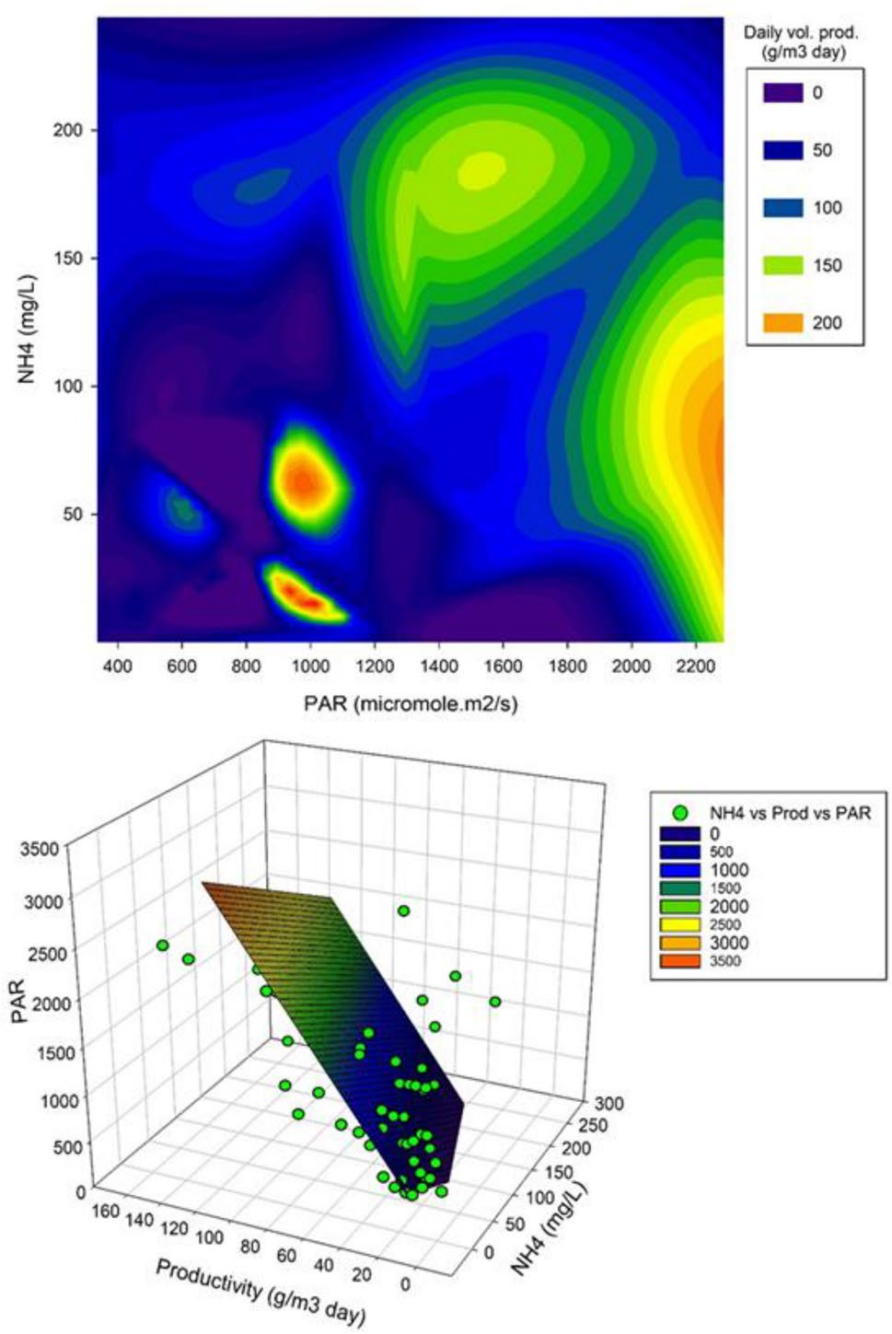
Effect of illumination and ammonia concentration in daily volumetric productivity. **a** Contour plot from obtained data obtained in Table 2. **b** Regression plot derived from the analysis of variables (*R*^2^ = 80%). Green data points represent individual information used for analyses. Illumination is expressed as Photosynthetic Irradiance Radiation (PAR) and ammonia concentration is in mg L^−1^. Illumination used the value of 4.57 to convert Watts m^−2^ to μmol m^−2^ s^−1^ and vice versa

Figure 8 showcases the effect of PAR and NH_4_^+^ concentrations on biomass productivity. It can be seen that the highest irradiation and low to medium ammonia concentrations produced the highest volumetric productivities. The high productivities depicted at low ammonia concentrations in Fig. 8a are considered to be an artefact. Overall, reported conditions are well represented in the response surface plots, about 94% of reports analysed use low to medium–high ammonium concentrations (50–170 mg L^−1^) and low to medium–high illumination values (430–1300 Watts m^−2^), with an average volumetric productivity between 50 and 100 g m^−3^ day^−1^.

### Validation of prediction factors

**Fig. 9:**
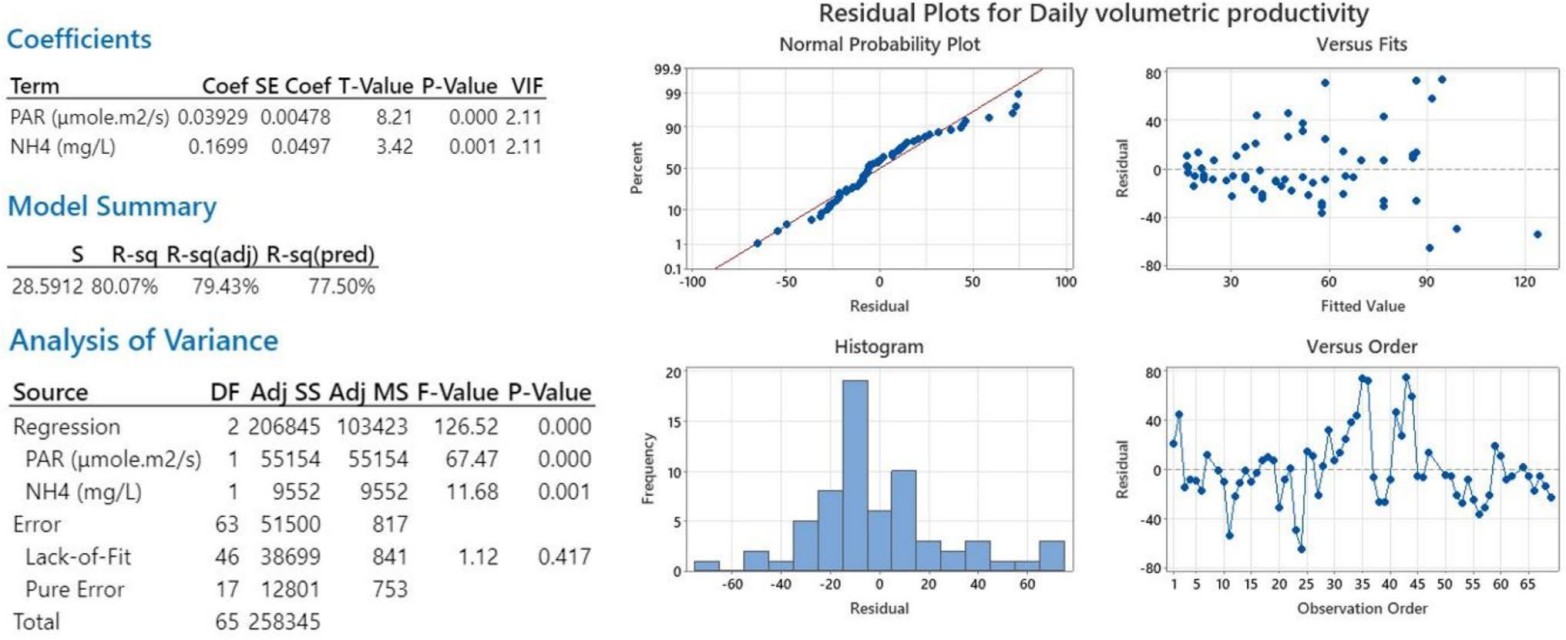
Residual plots from multiple regression analysis for daily productivity

Validation was conducted via the analysis of residual plots obtained from the correlation equation (Fig. 9); it can be seen that they follow a normal distribution. The normal probability plot shows the correlation of data according to the regression performed. Residual vs. fitted value shows that values are randomly distributed. The histogram shows a normal bell shape. Residual vs. observation order shows an aleatory order. According to the coefficients table displayed in Minitab, the value of the variance inflation factor (VIF) for significant variables was < 5 indicating no multicollinearity. To achieve this, it was necessary to perform several prior analyses to eliminate 12 insignificant or dependent correlated variables. As a result, it can be assumed that the regression model obtained using the two main variables fits the data well (NH_4_^+^ and PAR).

### HRAP vs. other microalgae cultivation systems

The two major microalgal cultivation systems for wastewater treatment are classified as open and closed (Chew et al. 2018; González-Camejo et al. 2021). There is also a third cultivation mode that uses both open and closed systems, known as hybrid systems (Cai et al. 2013; Razzak et al. 2017). Open systems are generally cultivated outdoors, whilst closed systems are usually cultivated indoors, although several studies report closed systems that are exposed to sunlight and ambient temperature (Molazadeh et al. 2019). At the pilot scale, open systems such as raceway reactors are presented as a viable technology for microalgae wastewater treatment, as they offer easy maintenance, higher operating volumes, and lower energy consumption, producing a similar microbial growth rate than closed systems, making them economically attractive (Morillas-España et al. 2021; Nordio et al. 2023). Our study aligns with literature that reports solar radiation and temperature to largely determine the success of pilot-scale open systems using high-capacity ponds. However, both variables are dependent on each other; therefore, only one can be used to predict productivity. It should be noted that open pond cultures are subject to inherent factors such as easy contamination, rapid water evaporation, and large surface occupancy. In addition, the stability of microalgal cultures and biomass yields may be affected by their susceptibility to contamination by competing organisms or predators such as rotifers (Kim et al. 2017; Jebali et al. 2018; Ma et al. 2018; Galès et al. 2019).

Contamination in hybrid systems can be significantly reduced compared to open systems. Hybrid systems create a two-staged growing cycle that can generate microalgal biomass in a rapid and controlled manner, which is then transferred to an open reactor. Hybrid systems include the plate reactors, tubular reactors, ring reactors, and airlift plate reactors. Here, photobioreactors are used to produce inoculum under controlled conditions, which are used for the cultivation of algae in open ponds, to reduce pollution problems and maintain species dominance (Cai et al. 2013). Recently, De Assis et al. (2020) proposed the use of biofilm reactors (BR) as an algal biomass production and harvesting unit, coupled with a HRAP, as a wastewater treatment unit. Under this approach, biomass production was achieved about 2.6 times higher than the conventional system without BR, and the harvesting efficiency of the hybrid system was 61%, compared to 22% obtained with the conventional system. The photobioreactor promoted an increase in density (~ 1,011 org m^−2^) and diversity of microalgae in the hybrid system and did not interfere with wastewater treatment. Economic analyses of the application of photobioreactors in hybrid systems are needed to enable their large-scale implementation.

## Conclusions

A multivariate analysis can assist in identifying key growth parameters within a series of environmental and operational factors. Correlation and multiple regression analyses undertaken suggest that among all parameters reported that influence biomass production, the key parameters are NH_4_^+^ and illumination (PAR). The content of NH_4_^+^ can be manipulated and controlled in reactors by using wastewater with a moderate ammonium-nitrogen content. Using compounds such as swine, poultry wastewater or digestate of food waste increases final biomass concentrations but does not necessarily result in an increased productivity. The production of microalgal biomass at a large scale using wastewater has a great synergistic potential as HRAP can remove 90%, 70%, and 50% of the NH_4_, COD, and PO_4_ content, respectively. With respect to the systems used for cultivation, HRAP are still preferred despite their low productivity, mainly due to the low construction and operation costs. A strategy to implement for these open systems is to select an ideal location and cultivation season and create a hybrid growing system. Further research is needed on hybrid culture systems to dictate how a closed photobioreactor can improve high-rate algal pond performance on a larger scale. Scaling up algal cultures from laboratory to outdoor production is still a challenge because it not only depends on operational and environmental factors but also requires the maintenance of biological cultures for long periods. For example, microbial-specific growth rates halved in pilot scale studies versus lab experiments indicating a scaling-up hotspot. This could be overcome through the construction of an adequate microalgae-bacteria consortia, provision of maximum solar illumination, and possibly enabling CO_2_ supply. Maintaining a balance between limiting carbon and nutrient sources such as NH_4_ and CO_2_ of microalgae (C:N:P) is imperative. The on-line monitoring of pH is recommended so that potential problems can be early detected, improving culture stability and avoiding culture collapse.

Ultimately, cultivation of microalgae in wastewater remains an emerging field in which careful considerations should be cost-effectively implemented to sustain long-term cultivations and wastewater treatment. Future studies are recommended to systematically compare different pilot-scale reactor designs and their associated process economics.

### Supplementary Information

Below is the link to the electronic supplementary material.

Supplementary file1 (XLSX 45.8 KB)

## Data Availability

Datasets can be found in the attached supplementary material, further information can be made available upon request.
